# Partitioning of Respiration in an Animal-Algal Symbiosis: Implications for Different Aerobic Capacity between *Symbiodinium* spp.

**DOI:** 10.3389/fphys.2016.00128

**Published:** 2016-04-18

**Authors:** Thomas D. Hawkins, Julia C. G. Hagemeyer, Kenneth D. Hoadley, Adam G. Marsh, Mark E. Warner

**Affiliations:** College of Earth, Ocean and Environment, School of Marine Science and Policy, University of DelawareLewes, DE, USA

**Keywords:** cnidarian-dinoflagellate symbiosis, zooxanthellae, citrate synthase, mitochondria, reef coral, *Exaiptasia pallida*, *Aiptasia*, CZAR

## Abstract

Cnidarian-dinoflagellate symbioses are ecologically important and the subject of much investigation. However, our understanding of critical aspects of symbiosis physiology, such as the partitioning of total respiration between the host and symbiont, remains incomplete. Specifically, we know little about how the relationship between host and symbiont respiration varies between different holobionts (host-symbiont combinations). We applied molecular and biochemical techniques to investigate aerobic respiratory capacity in naturally symbiotic *Exaiptasia pallida* sea anemones, alongside animals infected with either homologous ITS2-type A4 *Symbiodinium* or a heterologous isolate of *Symbiodinium minutum* (ITS2-type B1). In naturally symbiotic anemones, host, symbiont, and total holobiont mitochondrial citrate synthase (CS) enzyme activity, but not host mitochondrial copy number, were reliable predictors of holobiont respiration. There was a positive association between symbiont density and host CS specific activity (mg protein^−1^), and a negative correlation between host- and symbiont CS specific activities. Notably, partitioning of total CS activity between host and symbiont in this natural *E. pallida* population was significantly different to the host/symbiont biomass ratio. In re-infected anemones, we found significant between-holobiont differences in the CS specific activity of the algal symbionts. Furthermore, the relationship between the partitioning of total CS activity and the host/symbiont biomass ratio differed between holobionts. These data have broad implications for our understanding of cnidarian-algal symbiosis. Specifically, the long-held assumption of equivalency between symbiont/host biomass and respiration ratios can result in significant overestimation of symbiont respiration and potentially erroneous conclusions regarding the percentage of carbon translocated to the host. The interspecific variability in symbiont aerobic capacity provides further evidence for distinct physiological differences that should be accounted for when studying diverse host-symbiont combinations.

## Introduction

Symbioses in which one partner (the symbiont) resides within the cells of a larger host organism to form a “holobiont” (used here as the host-symbiont unit) are widespread and contribute significantly to the success of some important biological groups (Douglas, 2010). One well-studied symbiosis involves cnidarians and photosynthetic dinoflagellates (genus *Symbiodinium*). Symbiotic cnidarians benefit significantly from the organic carbon generated by algal symbiont photosynthesis, while the algae benefit from access to host-derived nutrients (Muscatine, 1990; Muller-Parker and D'Elia, 1997; Yellowlees et al., 2008; Davy et al., 2012). This nutritional exchange underpins the success of scleractinian corals and, therefore, supports the high productivity and biodiversity of coral reefs (Muscatine and Porter, 1977; Muller-Parker and D'Elia, 1997). Despite much research effort, we lack a full understanding of metabolic interactions between the two partners (see Davy et al., 2012 for review) and how these interactions vary between different cnidarian-*Symbiodinium* associations (Steen and Muscatine, 1984; Hoegh-Guldberg and Smith, 1989; Verde and McCloskey, 1996; Stat et al., 2008; Starzak et al., 2014; Leal et al., 2015).

In optimal environments, intracellular *Symbiodinium* can provide a host with a significant portion of its daily respiratory carbon requirements (Muscatine et al., 1981; Falkowski et al., 1984; Muscatine, 1990; Verde and McCloskey, 1998; Muller-Parker and Davy, 2001 for review). However, estimates of how much photosynthetically fixed carbon is provided to the host and, implicitly, how much is retained by the algal symbionts, have depended largely on the use of two methods. The first measures photosynthetic fixation of ^14^C- or ^13^C-bicarbonate and its subsequent translocation into host tissues (Trench, 1971; Hofmann and Kremer, 1981; Muscatine et al., 1984; Gattuso et al., 1993; Davy and Cook, 2001; Hughes et al., 2010; Tremblay et al., 2012, 2014; Hoadley et al., 2015; Kopp et al., 2015). The second technique, known as the “growth rate method,” relies on measurements of light- and dark oxygen fluxes alongside estimations of *in-hospite* symbiont growth rates and carbon contents (usually inferred from mitotic indices and cell volumes, respectively) to estimate the contribution of zooxanthellae (= *Symbiodinium*) to animal respiration (commonly abbreviated as CZAR) (Muscatine et al., 1981, 1983; McCloskey and Muscatine, 1984). This method has been applied more widely than have the ^14^C or ^13^C methods, presumably due to the relative ease with which oxygen fluxes can be measured (Fitt et al., 1982; Muller-Parker, 1984; Muscatine et al., 1984; Steen and Muscatine, 1984; Davies, 1991; Davy et al., 1996; Verde and McCloskey, 1996; Fitt and Cook, 2001; Grottoli et al., 2006; Starzak et al., 2014). However, both methods have specific limitations. The ^14^C and ^13^C methods do not account for the loss of activity from the system as CO_2_, or for the recyclingof respiration-derived CO_2_ by symbiont photosynthesis. These can cause errors in calculation of gross carbon uptake rates. The growth rate method makes two assumptions: (1) rates of host- and symbiont respiration are equivalent in the light and in the dark; and (2) as O_2_ consumption reflects the combined respiratory activities of animal host and algal symbiont, the proportions of host- and symbiont respiration reflect their relative biomass (designated ß and 1-ß, respectively) (Muscatine et al., 1981, 1983). Photorespiration and light-enhanced respiration in *Symbiodinium* (Burris, 1977; Tytler and Trench, 1986; Suggett et al., 2008; Schrameyer et al., 2014), and the stimulatory effect of irradiance on holobiont respiration (Edmunds and Davies, 1988; Harland and Davies, 1995; Anthony and Hoegh-Guldberg, 2003) call the first assumption into question, and testing assumption (2) has proven difficult. To date, estimates of algal symbiont respiration *in hospite* have generally relied on regression analyses of algal cell density as a predictor of holobiont respiration (Jacques et al., 1983; Hoegh-Guldberg and Smith, 1989; Hoogenboom et al., 2010), or on direct, *in-vitro* assessments of freshly isolated or cultured algal symbionts (Dustan, 1982; Muller-Parker, 1984; Fitt and Cook, 2001; Hoogenboom et al., 2010; Al-Sofyani and Floos, 2013). These approaches are not satisfactory, since the first assumes no effect of symbiont density on host respiration, and the second is likely confounded by the respiratory activities of residual host material and substantial physiological changes when *Symbiodinium* are removed from the host (see Goiran et al., 1996, 1997; Wang et al., 2011).

Aerobic respiration in eukaryotes occurs primarily in mitochondria; organelles that generate ATP through oxidative phosphorylation using tricarboxylic acid (TCA) cycle-derived NADH (Berg et al., 2002). In symbiotic cnidarians, the regulation of mitochondrial integrity and associated cell-signaling pathways is a component of their response to acute abiotic stressors such as elevated temperature (Dunn et al., 2012; Hawkins et al., 2013, 2014; Paxton et al., 2013; Lutz et al., 2015). Moreover, adjustments to mitochondrial function might be a general mechanism by which marine species acclimatize to a changing environment (Guderley and Johnston, 1996; Shama et al., 2014; Dixon et al., 2015). Given its importance to aerobic metabolism, it is surprising that mitochondrial activity has received relatively little attention as a useful marker of respiration in the cnidarian-*Symbiodinium* association. However, mitochondrial function, density, and integrity are commonly used markers of aerobic respiration in mammals (e.g., Srere, 1969; Wu et al., 1999; Spinazzi et al., 2011). For instance, analysis of mitochondrial genome copy number (relative to that of the nuclear genome) can be used to estimate mitochondrial density (Wu et al., 1999; Moraes, 2001; Miller et al., 2003; Ballard and Melvin, 2007; Urschel and O'Brien, 2008; Qiu et al., 2013). Additionally, the activity of mitochondrial electron transport complexes and of the TCA-cycle rate-limiting enzyme citrate synthase (abbreviated as CS hereafter) are useful correlates of respiratory activity in planktonic (Båmstedt, 1980; Clarke and Walsh, 1993; Thuesen and Childress, 1994; Torres et al., 1994; Kawall et al., 2001; Bellucci, 2004; De Wit et al., 2015) and benthic marine invertebrates (Marsh et al., 1999; Pace et al., 2006). Mitochondrial enzyme activity has been measured in corals and sea anemones (Sarkissian and Boatwright, 1975; Gattuso et al., 1993; Agostini et al., 2013; Henry and Torres, 2013; Rivest and Hofmann, 2014) but never, to our knowledge, with the specific aim of elucidating the partitioning of respiration between host and symbiont in an intact symbiosis.

We sought to investigate variability in respiratory function in the cnidarian-dinoflagellate symbiosis using a natural population of the sea anemone *Exaiptasia pallida* (= *Aiptasia pallida*, Grajales and Rodriguez, 2014) alongside anemones re-infected with either natural (homologous) or introduced (heterologous) *Symbiodinium* species. Initially, we developed a quantitative PCR (QPCR) assay to determine host mitochondrial genome copy number cell^−1^ (the ratio of host mitochondrial DNA [mtDNA] to nuclear DNA [nucDNA], henceforth referred to as “mtDNA/nucDNA”). We hypothesized that mtDNA/nucDNA is a significant predictor of protein-normalized mitochondrial citrate synthase enzyme activity and holobiont respiration rate, and thus reflects tissue mitochondrial density. Secondly, we hypothesized that activities of host- and symbiont mitochondrial citrate synthase enzyme are significant predictors of holobiont respiration rate. Due to the potential for inorganic nutrient- and carbon-limitation at high symbiont number (Cook et al., 1988; Weis, 1993), we predicted that *Symbiodinium* densities (per mg host protein) would correlate negatively with *Symbiodinium* specific CS activity. We also predicted that the inverse would be true for host specific CS activity, which might be expected to increase with symbiont density due to the increased availability of photosynthetically-fixed carbon (Agostini et al., 2013). Finally, to test assumption (2) of the CZAR calculation (Muscatine et al., 1981, 1983), we hypothesized that the partitioning of total holobiont CS activity between host and symbiont is approximately equal to their biomass ratio, and that this relationship holds across different host-symbiont combinations.

## Materials and methods

### Anemone husbandry, *Symbiodinium* removal, and re-infection

Specimens of *E. pallida* were collected from Key Largo, FL, USA in August 2014 and donated by Dr. William Fitt. Anemones were maintained in 15-L tanks supplied with constantly running natural seawater (1-micron-filtered and UV-sterilized, 26°C, 1 L min^−1^ flow rate). Irradiance (100 μmol photons m^−2^ s^−1^) was provided on a 12 h:12 h light:dark cycle (lights on at 06:00) provided by cool white LEDs (Cree XP-G2; LED Supply, Randolph, VT, USA). Aposymbiotic *E. pallida* were obtained by subjecting a subset of anemones (*n* > 200) to a menthol treatment (Wang et al., 2012; Matthews et al., 2015), with dark-incubation used instead of the photosynthetic inhibitor diuron. Aposymbiosis was confirmed by the complete absence of *Symbiodinium* using a fluorescence microscope [EVOS system, ThermoFisher (Life Technologies), Waltham, MA, USA; excitation: 628 ± 20 nm, emission: 692 ± 20 nm]. Aposymbiotic animals were maintained in a 30-L aquarium in the dark, and checked monthly for the presence of algal symbionts.

Novel *E. pallida-Symbiodinium* associations were established by exposing aposymbiotic anemones to one of two different *Symbiodinium* species (~1000 cells mL^−1^ in 500 mL seawater) according to the methods of Hoadley et al. (2015). One group (*n* > 30 animals) was incubated with a homologous, monoclonal *Symbiodinium* culture established from *Symbiodinium* originally isolated from the same *E. pallida* population and maintained in semi-continuous growth in f/2-Si culture media (Guillard, 1973) for period of at least 1 year. Another set of anemones (*n* > 30) was exposed to a heterologous culture of *Symbiodinium minutum* (ID: Pk702) originally isolated from the octocoral *Plexaura kuna*. *Symbiodinium* were genetically identified by amplifying and sequencing the internal transcribed spacer-2 (ITS2) region of nuclear ribosomal DNA (LaJeunesse, 2002). In addition, culture Pk702 was confirmed as *S. minutum* through the PCR-amplification and sequencing of the *B7Sym15* microsatellite locus (Pettay and LaJeunesse, 2007).

Algae were noted as ITS2-types A4 and B1 for the homologous symbiont and *S. minutum* isolates, respectively. Newly symbiotic animals were maintained in separate flow-through tanks for at least 3 months before any physiological analysis was undertaken. A third group of aposymbiotic anemones (*n* = 24) were placed under lights for 8 weeks without being exposed to any *Symbiodinium* cells. These anemones remained aposymbiotic (as confirmed by fluorescence microscopy). To confirm symbiont genetic identity in re-infected animals, *Symbiodinium* DNA was extracted from 5 to 10 anemones per group, and the ITS2 region was amplified as described by LaJeunesse (2002). In all instances, anemones contained the appropriate ITS2-type. Animals were fed weekly with freshly hatched *Artemia* nauplii and, to reduce the effect of trophic status on physiological variables, sampling was conducted 3 days prior to feeding. Further, all sampling occurred between 09:00 and 15:00 to lessen the influence of diel periodicity in metabolic activity (Akimoto et al., 2005; Hoadley et al., 2011; Sorek et al., 2013).

### Measurements of respiration and photosynthesis as *In-vivo* oxygen flux

Respiration and photosynthesis of individual anemones [oral disk diameter >4 mm for natural *E. pallida* (*n* = 53), 4–6 mm for re-infected animals (*n* = 19 per *Symbiodinium* species)] was measured in sealed glass scintillation vials fitted with an internal stir bar and an oxygen sensitive optode (Fibox 4, PreSens Gmbh, Regensburg, Germany). Vials were immersed in a constant 26°C water bath and maintained in darkness for 15 min, during which time anemones relaxed and expanded their tentacles. Illumination was provided for 20 min at an irradiance of 200 μmol photons m^−2^ s^−1^ (slightly below saturating irradiance, and provided by the same LEDs described above), after which, the LEDs were turned off for a final 30-min period to allow measurement of steady-state dark respiration. Background O_2_ flux was determined using vials containing 1-μm-filtered, UV-sterilized seawater, and was found to be negligible. After respirometry assays, each anemone was transferred to a 2-mL screw-cap vial, snap-frozen in liquid nitrogen, and stored at −80°C. The volume of seawater in the chamber was measured, and rates of holobiont dark respiration were calculated as moles O_2_ consumed hour^−1^. Dark respiration rates were then subtracted from net photosynthetic rates (moles O_2_ produced hour^−1^ during the light-phase) to generate values for gross photosynthesis. Respiration- and gross photosynthesis rates were normalized to soluble animal protein and symbiont cell number, respectively (described below).

### Anemone processing and *Symbiodinium* density analysis

Unless otherwise indicated, reagents were obtained from Fisher Scientific (Pittsburgh, PA, USA) and all steps were carried out at 4°C or on ice. Anemones were thawed in their 2-mL screw cap vials and 0.6–1 mL ice-cold lysis buffer (25 mM Tris, pH 7.8, 1 mM EDTA, 10% glycerol [v/v]) was added to each vial. Anemones were then homogenized in a chilled bead-beater (FastPrep®-24, MP Bio, Santa Ana, CA, USA) for 60 s at a speed of 6 m s^−1^ with a 5-mm-diameter stainless steel bead. The homogenate was inspected visually (100 × magnification) to confirm anemone tissue disruption and *Symbiodinium* cell integrity. A 100-μL sample was removed, fixed with 5 μL glutaraldehyde (8% [w/v] stock solution in water), and stored at 4°C in the dark for later quantification of *Symbiodinium* cell densities. The remaining homogenate was centrifuged at 3000 × g for 30 s. Two hundred microliters of the supernatant were removed for animal DNA extraction (see below) and the pellet was re-suspended. The sample was then centrifuged for 5 min at 700 × g to separate the *Symbiodinium* cells from the remaining anemone material. The *Symbiodinium* pellet was immediately snap-frozen in liquid nitrogen and transferred to a −80°C freezer. The supernatant (“anemone fraction”) was centrifuged at high speed (16,100 × g, 20 min) to remove particulates, and aliquots of the clear supernatant were snap-frozen and stored at −80°C.

In order to remove residual animal protein, *Symbiodinium* pellets were thawed, re-suspended in 1 mL ice-cold wash buffer (as lysis buffer above, but with the addition of 0.01% [v/v] Triton X-100), and centrifuged for 5 min at 700 × g. The supernatant was discarded and the pellet re-suspended in fresh ice-cold wash buffer. This procedure was repeated four times, after which the supernatant was clear and the pellet dark brown, with little evidence of contaminating anemone material. *Symbiodinium* cells were finally re-suspended in 300 μL ice-cold lysis buffer in a clean 1.5-mL tube containing a 200-μL-volume of 0.5-mm-diameter acid-washed glass beads. Cells were lysed in a chilled bead-beater (see above) for 3 min at a speed of 6.5 m s^−1^. The lysate was inspected visually (100 × magnification), before centrifugation to remove particulates (16,100 × g, 20 min). Aliquots of the supernatant were then snap-frozen in liquid nitrogen and stored at −80°C.

Total soluble anemone and *Symbiodinium* protein were determined using a linearized Bradford assay (Ernst and Zor, 2010). To test the effectiveness of the algal washing steps described above, a standard curve was constructed by spiking 12 *Symbiodinium* pellets from similar-sized anemones (in triplicate) with 24–1200 μg anemone protein “contamination” originating from crude homogenates that had been gently centrifuged (500 × g for 5 min) to remove algal cells. Algal pellets were then washed and a 50-μL sample was removed and fixed for cell counts. The remaining cells were lysed as described above. *Symbiodinium* protein content was then measured for each pellet, normalized to cell number (see below), and plotted against the respective amount of anemone material added.

*Symbiodinium* densities were quantified using an Improved Neubauer hemocytometer and a fluorescence microscope to visualize cells' chlorophyll *a* fluorescence (see above). Field of view was determined using the EVOS operating software (4 × objective), and cells were counted using the “Analyze Particles” tool in ImageJ (NIH, Bethesda, MD, USA). At least 6 independent images were analyzed for each sample, and cell numbers were normalized to anemone protein content.

### Biochemical analysis of mitochondrial function

Anemone and *Symbiodinium* aerobic capacity was quantified as the activity of the TCA cycle rate-limiting enzyme citrate synthase (CS), measured according the methods of Srere (1969) modified for use with small marine invertebrates. All samples were analyzed within 1 month of freezing. Representative results of assay optimization and validation procedures are provided in ESM Figure S1. Briefly, 20 μL of thawed anemone or *Symbiodinium* supernatant (diluted to yield 2–6 μg of protein) was added in triplicate to wells in a 96-well plate (Greiner Bio-One, Monroe, NC, USA) alongside triplicate blanks (20 μL lysis buffer) and positive controls [20 μL citrate synthase (1 U mL^−1^ in lysis buffer; Sigma-Aldrich, St. Louis, MO, USA)]. One hundred and seventy microliters of assay buffer (111 mM Tris, pH 7.8, 0.11% [v/v] Triton X-100) containing 294 μM 5,5′-dithiobis-(2-nitrobenzoic acid) (DTNB; Sigma-Aldrich, see above) and 588 μM acetyl-coenzyme A (Sigma-Aldrich, see above) were then added to all wells. DTNB stock solutions (5 mM) were made fresh in 0.1 mM Tris buffer, pH 8.0. Acetyl-coenzyme A solutions were prepared at a concentration of 12.35 mM in distilled water, stored in aliquots at −80°C, and used within 6 months.

To control for non-CS-specific reaction products following the addition of assay buffer, baseline absorbance (λ = 412 nm) was recorded for 3 min using a microplate reader (Fluostar Omega, BMG, Cary, NC, USA) maintained at a temperature of 26°C. The CS-catalyzed reaction was initiated by adding 10 μL oxaloacetate to each well (OA; Sigma-Aldrich, see above; 12 mM stock solution prepared fresh in distilled water and stored on ice), giving a final concentration of 600 μM OA. Sample absorbance was monitored at 412 nm for a further 3 min, and CS enzyme activity was derived using the following equation:

$$\begin{array}{l} CS\;Specific\;Activity\left(U\;{mg}^{-1}\right)\\ =\frac{\left({△412}_{OA}-\;{△412}_{blank}\right)\times V_{reaction}\times D}{13.6\;\times L\;\times \;V_{sample}\times P} \end{array}$$

Where Δ412 is the linear rate of change in 412-nm absorbance prior to and after the addition of OA, V is the volume (mL), D is the sample dilution factor, 13.6 (mM^−1^ cm^−1^) is the 412-nm extinction coefficient for the reaction product, L is the optical path-length (cm) and P is the sample protein concentration (mg mL^−1^). Total *Symbiodinium* and animal CS activities were calculated as the product of the respective specific activity and total protein contents.

### Quantification of anemone mitochondrial copy number

A plasmid-cloning and quantitative-PCR approach was used to determine mtDNA/nucDNA in the natural *E. pallida* population. Since DNA extraction method can influence the apparent mtDNA/nucDNA ratio (Andreu et al., 2009; Guo et al., 2009), anemone genomic DNA was extracted using two independent methods. For some animals (*n* = 33), DNA was extracted using a Wizard® Genomic DNA Extraction kit (Promega Corporation, Madison, WI, USA). Briefly, the 200-μL host homogenate was initially mixed with 400 μL Wizard® nuclei lysis buffer. Samples were incubated with 0.1 mg mL^−1^ proteinase K for 2 h at 60°C (vortexed every 20 min). Proteins were removed by precipitation with 360 μL 9 M ammonium acetate, 20-min incubation at 4°C and centrifugation at 15,000 × g for 5 min. Six hundred microliters of supernatant were aspirated and transferred to a clean tube containing 700 μL isopropanol and 25 μL 3 M sodium acetate. DNA was left to precipitate on ice for 20 min before centrifuging (15,000 × g for 5 min). The DNA pellet was washed with ethanol, air-dried under sterile conditions at 30°C, and finally dissolved in 50 μL nuclease-free water (BioExpress, Kaysville, UT, USA). DNA from a second group (*n* = 20) was extracted and purified using a QiaAmp DNA Mini kit (Qiagen, Germantown, MD, USA), with the 200-μL aliquot of animal homogenate initially mixed with 200 μL “Buffer AL” and 20 μL Qiagen Proteinase K. DNA was purified following the manufacturer's instructions—albeit with an additional column-wash with “Buffer AW2”—and eluted in 50 μL “Buffer AE.” DNA concentration was measured using a Quant-iT PicoGreen® assay (ThermoFisher [Invitrogen], Waltham, MA, USA) and purity was determined spectrophotometrically as the 260/230 nm and 260/280 nm absorbance ratios (NanoDrop®, ThermoFisher, Waltham, MA, USA). The 260/280 nm ratio was consistently >1.8, but the 260/230 nm ratio was variable, particularly in samples purified using the Wizard® Promega kit (range = 1.4–2.2), however, this had a negligible effect on the efficiency of subsequent QPCR reactions (see ESM, Figure S2A).

#### Primer design and standard curve construction

Oligonucleotide primers (Table 1) were designed using web-based software (PrimerQuest®, Integrated DNA Technologies Inc., Coralville, IA, USA) for two mitochondrial DNA sequences and one nuclear DNA sequence. Mitochondrial sequences were: (1) 101 bp of the cytochrome *c* oxidase subunit 1 gene (*CO1*); and (2) 144 bp of the ATP-sythase subunit 6 gene (*ATP6*). The nuclear DNA sequence was 89 bp of the nuclear gene eukaryotic translation elongation factor 1-alpha (*EF-1-*α), which is present as a single copy in *E. pallida* (Baumgarten et al., 2015). Template sequences for mitochondrial targets were obtained from the mitochondrial genome of *Aiptasia pulchella* [*CO1*: nucleotides 15,388–17,860 in GenBank Acc. No: NC_022265, (http://www.ncbi.nlm.nih.gov/gene/16792268); *ATP6*: nucleotides 19086–19775 in NC_022265, (http://www.ncbi.nlm.nih.gov/gene/16792265)]. *E. pallida* EF-1-α was identified by BLAST-aligning an *EF-1-*α sequence from the *Nematostella vectensis* genome (Putnam et al., 2007) against an *A. pallida* expressed sequence tag library (AiptasiaBase; Sunagawa et al., 2009). BLASTX analysis of the best hit (AiptasiaBase “contig 642,” *E* = 0) against the *A. pallida* genome (Baumgarten et al., 2015; http://aiptasia.reefgenomics.org/) strongly matched an EF-1-α protein (*E* = 0; Gene ID: gnl|BL_ORD_ID|13307, AIPGENE865 sp|P29520).

**Table 1:** **Primers used for the amplification of mitochondrial- and nuclear target genes in order to quantify host mitochondrial genome copy number (mtDNA/nucDNA ratio) in *Exaiptasia pallida***.

**Objective**	**Target**	**Size (bp)**	**Forward Primer (5′–3′)**	**Reverse Primer (3′–5′)**
TOPO® Cloning (standard curve)	*COI*	356	AGC CGT CAG AGA CAG TAA	CGT GAC CAA GCC CTA ATA AA
	*ATP6*	342	GGT TGT TAC GTT AGG CTT GT	ACT TGA ATA ACC GCC ACT
	*EF-1-α*	803	TTC TCT CTT ACA CCC TTG G	CTT GTC AGT GGG TCT CTT
QPCR	*COI*	101	GTC TCC CAG CCG GAA ATA AA	ACC ATT GTC AGC ATC TCT CG
	*ATP6*	144	CGT CTC GCC GCA AAT TTA TC	TGC GGC CTC TAG TAG ACT TA
	*EF-1-α*	89	AGC ACT GAG CCA CCA TAC AG	TTG GGT TAT AGC CGG TCT TC

*CO1, Cytochrome c oxidase subunit 1; ATP6, ATP synthase subunit 6; EF-1-α, Eukaryotic translation elongation factor 1-alpha. Annealing temperature was 60°C for all primer sets. Specific details of PCR conditions are provided in the accompanying Electronic Supplementary Information*.

Primer validation was undertaken using end-point PCR, with each reaction containing 20 ng anemone DNA in a 20-μL mix of 1 × Standard Mg-free PCR buffer, 0.25 U Taq DNA polymerase, 0.25 μM (*CO1* and *ATP6*) or 0.5 μM primers (*EF-1-*α), 2.5 mM MgCl2, and 0.25 mM dNTPs (reagents from New England Biolabs, Ipswich, MA, USA; primers from Integrated DNA Technologies, see above). Cycling conditions were 94°C for 2 min, followed by thirty cycles of 94°C for 15 s, 60°C for 30 s, and 72°C for 30 s, with a final elongation at 72°C for 10 min. Agarose gel electrophoresis confirmed a single PCR product for each primer set and, after product purification (ExoSAP-IT, Affymetrix, Santa Clara, CA, USA), amplicons were sequenced in both directions using the respective PCR primers (GeneWiz, South Plainfield, NJ, USA). Electropherograms were inspected visually to confirm the reliability of base-calling and sequences were compared to NCBI Genbank or *Aiptasia* genome databases. In all cases the sequences aligned most strongly with those used for primer design.

Additionally, a longer sequence of each of the three target genes (>300-bp, encompassing the respective QPCR target) was PCR-amplified (primers as in Table 1, PCR conditions as above) and cloned into bacterial plasmids using a TOPO TA Cloning® kit (Life Technologies, see above). Plasmids were then transformed into One Shot® Mach1^*TM*^ T1 phage-resistant chemically competent *Escherichia coli* (Life Technologies, see above). After 24 h bacterial growth in Luria-Bertani media at 37°C, plasmids were extracted and purified using a PureLink® Plasmid MiniPrep kit (Invitrogen, see above). Inserts were sequenced in both directions using M13R and M13(-21)F primers (Genewiz, South Plainfield, NJ, USA) to confirm the presence and correct orientation of the respective inserts. Plasmid DNA was then linearized with EcoR1 restriction endonuclease (Invitrogen, see above; ESM Figure S3) and quantified using a PicoGreen® assay. Linearized plasmids (encoding the respective QPCR target sequences) were used to construct a seven-level log_10_-dilution standard curve (10^7^–10^1^ copies per reaction, in triplicate) prior to each set of QPCR reactions. Sequences for QPCR amplicons and longer TOPO TA-cloned fragments are provided in the accompanying ESM.

#### QPCR analysis

Duplicate 2-μL aliquots of extracted DNA (5–10 ng μL^−1^) were added to a 18-μL reaction mix (SensiMix™ SYBR Hi-ROX; Bioline, Taunton, MA, USA) such that the final mix contained 0.25 μM *CO1* or *ATP6* primers, or 0.5 μM *EF-1-*α primer. Gene-fragments were amplified using an AB-7500 real-time QPCR system [ThermoFisher (Applied Biosystems), Waltham, MA, USA], with the following cycling conditions: 94°C for 10 min, followed by 40 cycles of 94°C for 15 s, 60°C for 1 min, and 72°C for 15 s. A melt-curve analysis (60–94°C in 0.3°C increments, 30 s per step) was carried out in order to detect non-specific amplification products. A single PCR product was detected in all cases, with a melting temperature within 1°C of the theoretical melting temperature of the sequenced amplicon as determined by a web-based tool (OligoCalc; Kibbe, 2007). Baseline values were determined automatically and threshold value was set manually at 0.04 (maintained across all samples and standards). Amplification efficiencies were 93–96% in all instances. The number of *CO1, ATP6*, and *EF-1-*α sequences per 20-μL reaction was determined by comparing mean C_t_ values for each sample to the respective log-dilution standard curve, and *CO1*/*EF-1-*α and *ATP6*/*EF-1-*α ratios were then calculated.

### Statistical analysis

Total anemone- and *Symbiodinium* protein contents were used to define the parameters ß and 1-ß, respectively (Muscatine et al., 1981), with the latter representing the proportion of *Symbiodinium* biomass relative to that of the holobiont (“symbiont biomass fraction”). Likewise, the algal contribution to holobiont CS activity was calculated (designated as the “symbiont respiration fraction,” abbreviated as R_Sym_). Predictive associations between symbiont density, host-, symbiont-, and total holobiont citrate synthase activity, host mtDNA/nucDNA ratio and total biomass, holobiont respiration, 1-ßand R_Sym_ were investigated using linear regression models [“lm()” function] in R v. 3.2.1 (R Development Core Team, 2015). Standardized residual plots were examined visually and residuals were tested for homoscedasticity and normality. Distributions and variances of standardized residuals were, in a few cases, not normal or homogeneous. Therefore, all regression analyses were bootstrapped to improve robustness and to provide estimates of 95% confidence intervals for regression parameters [“boot()” function; *n* = 2000 replications]. Where a multiple regression analysis was performed, co-linearity between predictors was examined using the “vif()” function in R package “car.” Associations where a predictor was not hypothesized *a priori* were analyzed using Pearson's correlation test. Comparisons between anemones re-infected with homologous ITS2-type A4 *Symbiodinium* or heterologous *S. minutum* were conducted using Student's *t*-tests. Additionally, differences in the relationship between 1-ß and R_Sym_ in the different holobionts were tested for using a multiple regression, with 1-ß and “holobiont” as independent predictors of R_Sym_. Assumptions of normality and homoscedasticity were assessed using Shapiro-Wilk- and Levene's tests, respectively. Non-normal or heteroscedastic data were log_10_-transformed prior to analysis with *t*-tests. Data that could not be adequately transformed were analyzed using non-parametric Wilcoxon Rank-Sum tests.

## Results

### Validation of biochemical and molecular techniques

**Figure 1:**
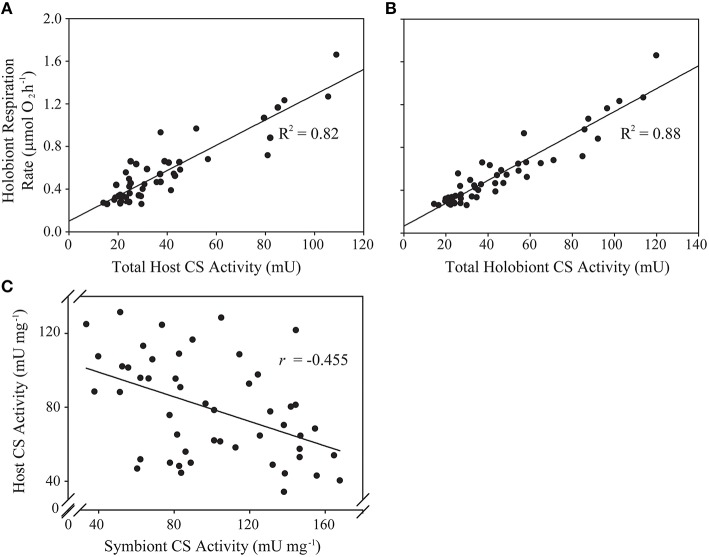
**Predictive and correlative associations between animal host- and algal symbiont citrate synthase (CS) activity and holobiont respiration rate in a natural population of *Exaiptasia pallida*. (A)** Linear regression analysis of total host CS activity (U) as a predictor of holobiont respiration rate (μmol O_2_ h^−1^); **(B)** Linear regression analysis of total holobiont CS activity (host + symbiont; U) as a predictor of holobiont respiration rate (μmol O_2_ h^−1^); **(C)** Pearson's correlation analysis of the relationship between host- and symbiont specific CS activities (U mg^−1^). All relationships are statistically significant at *p* < 0.001.

Total anemone citrate synthase (CS) activity was a significant predictor of holobiont respiration rate, explaining 82% of observed variance (Figure 1A, Table 2). *In-hospite Symbiodinium* CS activity was also a significant predictor of holobiont respiration (Table 2), although it explained less variation than did host CS (*R*^2^ = 0.70 *vs*. *R*^2^ = 0.82). The explained variance in holobiont respiration increased to 88% when total host and symbiont CS activities were combined (Figure 1B, Table 2).

**Table 2:** **Results of bootstrapped linear regression analysis (*n* = 2000 replications) of anemone host, algal symbiont-, and total holobiont citrate synthase (CS) activity (U) in *Exaiptasia pallida* as predictors of total holobiont respiration rates (μmol O_2_ h^−1^)**.

**Predictor**	**Value (95% CI)**	***t***	***p*(*t*)**	***F*(df)**	***p*(*F*)**
Total host CS activity	11.867 (9.50, 13.84)	14.849	<**0.0001**	220.484 (1, 48)	<**0.0001**
Intercept	0.100 (0.033, 0.173)	2.818	**0.007**		
Total symbiont CS activity	21.947 (18.34, 28.88)	9.032	<**0.0001**	81.570 (1, 48)	<**0.0001**
Intercept	0.306 (0.264, 0.362)	11.239	<**0.0001**		
Total holobiont CS activity	10.715 (9.27, 12. 43)	19.089	<**0.0001**	364.383 (1, 48)	<**0.0001**
Intercept	0.064 (-0.004, 0.118)	2.162	**0.036**		

*Bold values indicate statistical significance at p < 0.05*.

For the *Symbiodinium* CS samples artificially “spiked” with animal homogenates, the apparent protein content per *Symbiodinium* cell did not change significantly [Linear Regression; Predictor: amount of anemone protein added to sample, Dependent: *Symbiodinium* protein content per cell, *F*_(1, 11)_ = 0.49, *p* > 0.5, ESM Figure S2B]. Furthermore, there was no positive correlation between anemone and *Symbiodinium* specific CS activities (per mg protein), which would have been expected if CS-rich anemone material was contaminating the algal samples. Rather, a statistically significant negative correlation was apparent (Figure 1C; Pearson's correlation test: *r* = −0.455, *t* = −3.538, df = 48, *p* = 0.0009).

**Figure 2:**
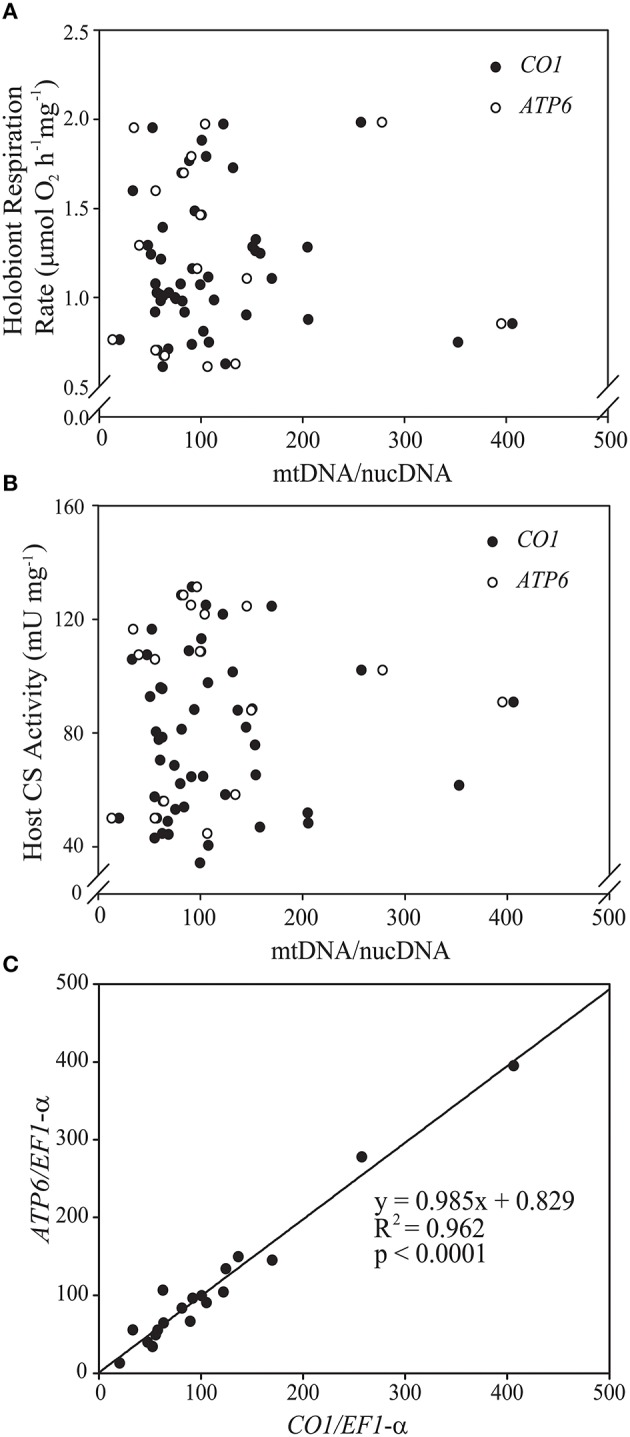
**Scatterplots of the relationships in *Exaiptasia pallida* between mitochondrial genome copy number (mtDNA/nucDNA ratio) and (A) holobiont respiration rate normalized to host protein content (μmol O_2_mg^−1^ h^−1^) and (B) host citrate synthase (CS) specific activity (U mg^−1^)**. Closed symbols represent values obtained using the mitochondrial cytochrome *c* oxidase subunit 1 (*CO1*) gene, while open symbols represent values obtained using the mitochondrial ATP-synthase subunit 6 (*ATP6*) gene. Copy numbers for both genes were normalized to that of eukaryotic translation elongation factor 1 alpha (*EF-1-*α), a single-copy nuclear gene in *E. pallida*. Neither of the relationships in **(A,B)** was statistically significant at *p* < 0.05. **(C)** Strong positive association between mtDNA/nucDNA as calculated using *CO1* and *ATP6* copy numbers normalized to *EF-1-*α.

In addition to analysis of CS activity, we tested whether the animal mitochondrial/nuclear genome ratio might be a useful indicator of mitochondrial density in *E. pallida*. The DNA extraction method had no effect on the mtDNA/nucDNA ratio (ESM Figure S2C). Therefore, data were pooled for subsequent analyses. Anemone mtDNA/nucDNA was highly variable (range = 20–300) and was not a significant predictor either of protein-normalized whole-organism respiration rate or anemone specific CS activity (Figures 2A,B). The very strong correlation between *CO1/EF-1-a* and *ATP6/EF-1-a* ratios (*R*^2^ > 0.95; Figure 2C) meant that this was the case regardless of which mitochondrial gene was used as the mtDNA target.

### Variability in respiratory function in *E. pallida*

**Figure 3:**
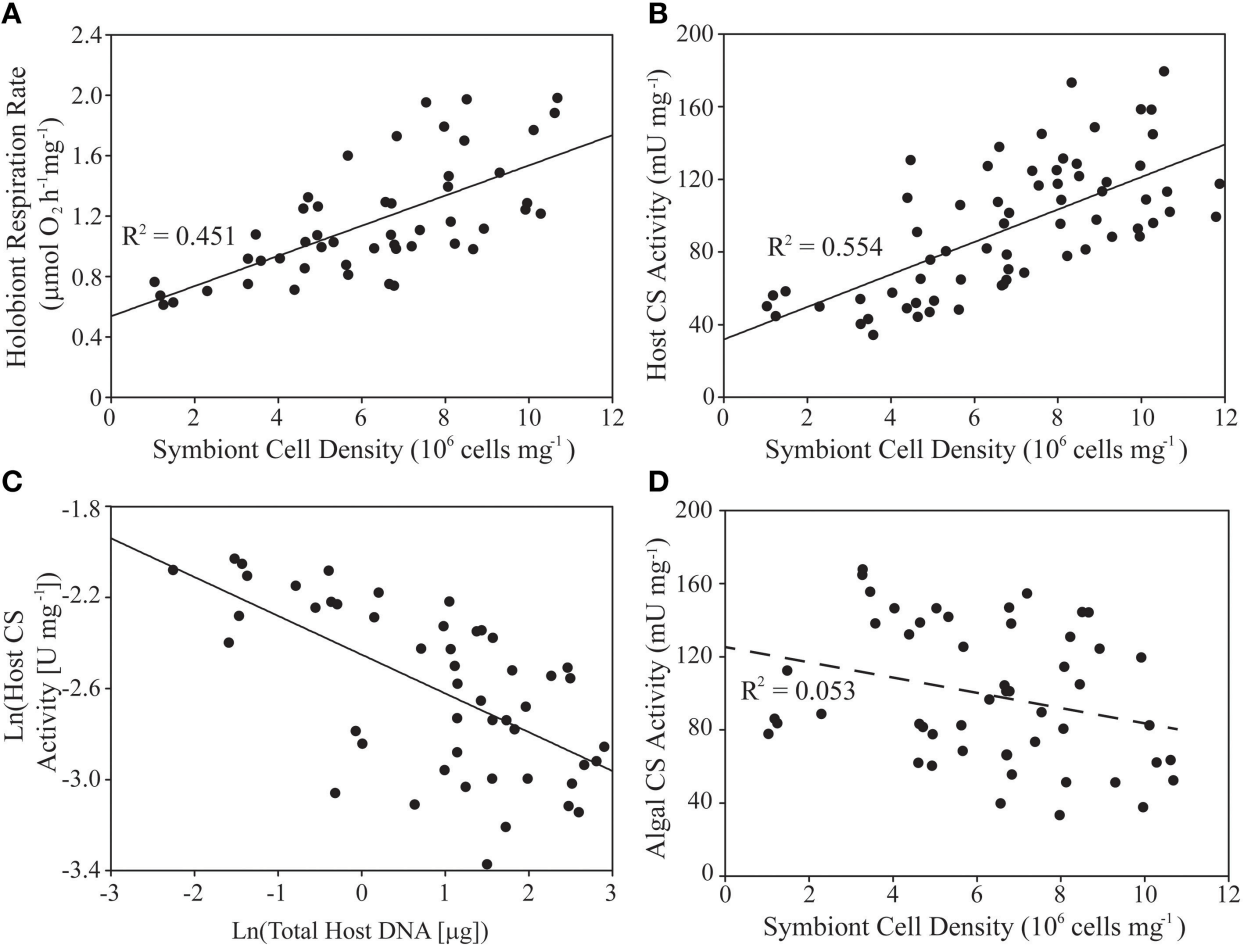
**Variability of holobiont respiration rate, animal host- and algal symbiont citrate synthase (CS) specific activities, host biomass, and symbiont density within a natural population of *Exaiptasia pallida*. (A,B,D)** Linear regression analysis of algal symbiont density (cells mg^−1^) as a predictor of **(A)** holobiont respiration rate normalized to host protein content (μmol O_2_mg^−1^ h^−1^), **(B)** host CS specific activity (U mg^−1^), and **(D)** symbiont CS specific activity (U mg^−1^). **(C)** Linear regression analysis of natural-log-transformed host biomass (mg protein) as a predictor of natural-log-transformed host CS specific activity (U mg^−1^). Relationships in **(A–C)** are statistically significant at *p* < 0.001 (**D**, *p* > 0.05). See text for details of multiple regression analysis of the data in **(B,C)**.

In naturally symbiotic *E. pallida*, holobiont respiration rate (mg host protein^−1^) and host CS specific activity were both positively associated with algal symbiont density (Figures 3A,B, Table 3). However, we also observed a negative log-linear relationship between host specific CS activity and anemone biomass (total animal DNA content; Figure 3C). Therefore, a multiple regression model was applied in order to parse the effects of these two covariates. Symbiont density alone explained 55.4% of variance in natural log-transformed host CS activity (see Table 3 for regression analysis parameters). Including total animal DNA (natural log-transformed) as a predictor increased the explained variance to 73.6% [*t*_biomass_ = −5.653, *p* < 0.0001; Full model: *F*_(2, 46)_ = 67.79, *p* < 0.0001]. Alone, total animal DNA explained 38.5% of variation in host specific CS activity [*t*_biomass_ = −5.574, *p* < 0.0001; Full model: *F*_(1, 47)_ = 31.07, *p* < 0.0001]. A weak negative association (statistically significant only for the y-intercept) was noted between symbiont CS specific activity and algal symbiont density (Figure 3D, Table 3).

**Table 3:** **Results of bootstrapped linear regression analysis (*n* = 2000 replications) of symbiont density per mg of host protein as a predictor of holobiont respiration rate normalized to host protein content (μmol O_2_ mg^−1^ h^−1^), and host- and symbiont specific citrate synthase (CS) activities (U mg^−1^)**.

**Dependent**	**Parameter**	**Value (95% CI)**	***t***	***P*(*t*)**	***F* (df)**	***p*(*F*)**
Holobiont respiration rate	Slope	0.100 (0.075, 0.125)	5.106	<**0.0001**	41.173 (1, 49)	<**0.0001**
	Intercept	0.537 (0.403, 0.668)	6.417	<**0.0001**		
Host CS activity	Slope	0.009 (0.007, 0.011)	7.993	<**0.0001**	63.89 (1, 67)	<**0.0001**
	Intercept	0.037 (0.01, 0.063)	3.747	**0.0004**		
Symbiont CS activity	Slope	-0.004 (-0.006, 0.000)	-1.940	0.058	3.745 (1, 49)	**0.0131**
	Intercept	0.125 (0.107, 0.165)	9.130	<**0.0001**		

*Bold values indicate statistical significance at p < 0.05*.

**Figure 4:**
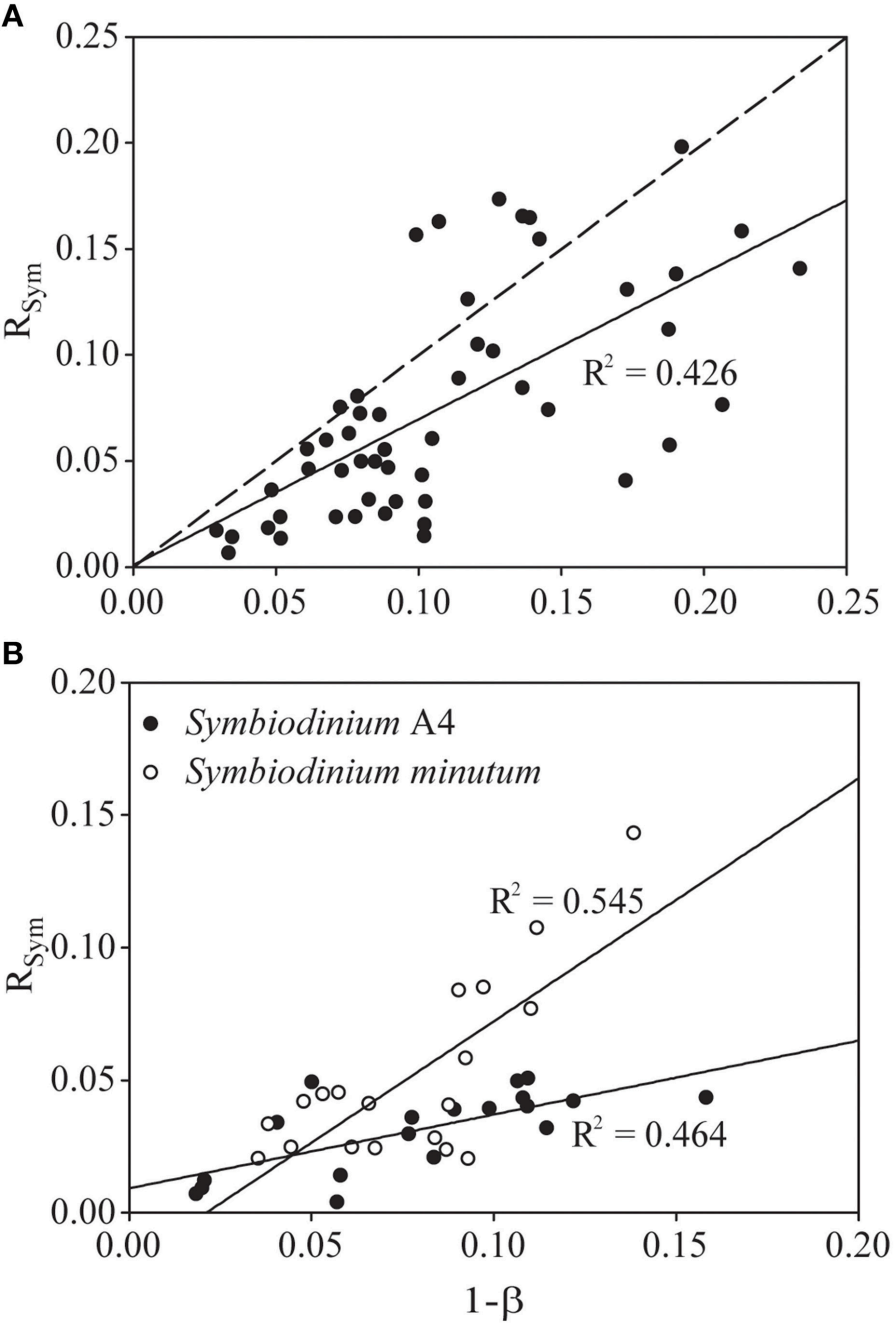
**Relationship between the proportion of total holobiont citrate synthase (CS) activity that is symbiont-derived (R_*Sym*_) and the contribution of symbiont biomass (measured as protein) to the total holobiont biomass (1-ß, see Muscatine et al., 1981) in (A) a natural population of *Exaiptasia pallida* and (B) two groups of *E. pallida* re-infected with either *Symbiodinium* type A4 or *Symbiodinium minutum***. The dotted line in **(A)** describes *y* = *x*. The solid lines describe the actual relationship between R_Sym_ and 1-ß in each group of animals, and are statistically significant at *p* < 0.001.

Analysis of CS enzyme activity as an indicator of aerobic capacity allowed us to estimate the partitioning of respiratory activity between host and symbiont and relate this to symbiont biomass. The symbiont biomass fraction (1-ß; as defined by Muscatine et al., 1981) ranged from 0.03 to 0.21 in our natural *E. pallida* population, with a median value of 0.09 (*n* = 53). These data are plotted against the symbiont-respiration fraction (R_Sym_) in Figure 4A, alongside a line with slope = 1 describing equality between 1-ß and R_Sym_. Concretely, any point above (or below) this line represents a holobiont with a higher (or lower) R_Sym_ than would be expected from the value of 1-ß. The slope of the fitted regression line was significantly different from unity [*F*_(1, 49)_ = 38.03, *p* < 0.0001; Slope = 0.689_[(95 *%CI*:0.475, 0.900), *t* = 6.167, *p* < 0.0001]_], which suggests that R_*Sym*_ and 1-ß are not equivalent in our naturally symbiotic *E. pallida* anemones.

**Figure 5:**
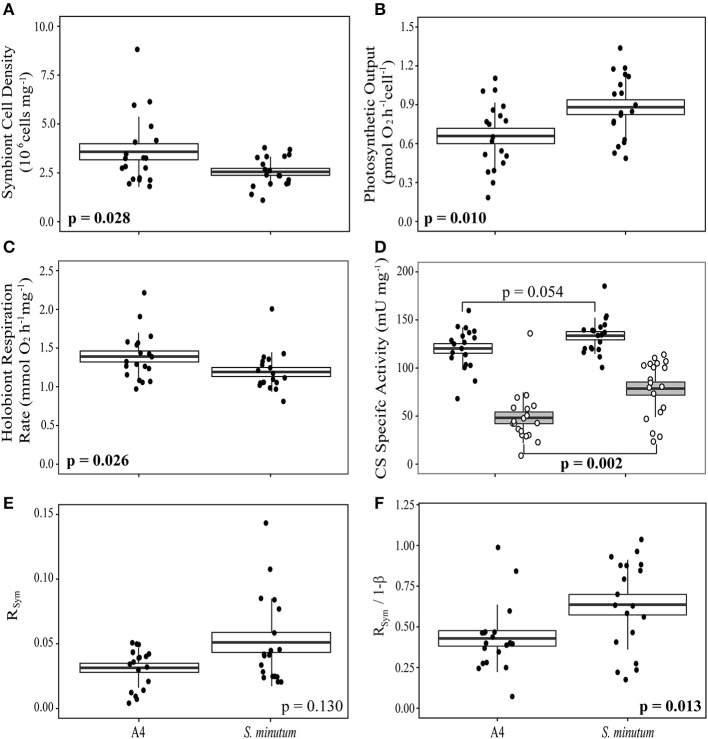
**Patterns of respiration and photosynthesis in two groups of *Exaiptasia pallida* experimentally re-infected with homologous *Symbiodinium* ITS2-type A4 or heterologous, ITS2-type B1 *S. minutum*, respectively. (A)** Symbiont cell densities (10^6^ cells mg^−1^); **(B)** Symbiont photosynthetic output (pmol O_2_ cell^−1^ h^−1^); **(C)** Holobiont respiration rate normalized to host protein content (μmol O_2_mg^−1^ h^−1^); **(D)** Host (white box, black markers) and symbiont (gray box, white markers) specific citrate synthase (CS) activities (mU mg^−1^); **(E)** The proportion of symbiont-derived CS activity relative to total holobiont CS activity (R_Sym_); **(F)** The relationship between R_Sym_ and symbiont biomass as a fraction of total biomass (1-ß; Muscatine et al., 1981). Boxes represent means (thick black line) ± 1 standard error, whiskers represent 1 standard deviation. Between-group comparisons for data in **(A–C,F)**, and host CS specific activity in **(D)**, were conducted using *t*-tests. Between-group comparisons for symbiont CS specific activity **(D)** and R_Sym_ **(E)** were conducted using Wilcoxon Rank-Sum tests.

In anemones separately re-infected with homologous and heterologous *Symbiodinium* species, we noted significant differences in symbiont density and holobiont respiration, both of which were higher in animals hosting the homologous *Symbiodinium* A4 (Figures 5A,B). *S. minutum* displayed a higher rate of photosynthetic activity per cell than did *Symbiodinium* A4 (Figure 5C), and this pattern was repeated in its CS specific activity, which was significantly elevated relative to that of *Symbiodinium* A4 (Figure 5D). Host CS specific activity did not differ between the two groups (Figure 5D), and no significant difference was observed in R_Sym_ (Figure 5E, Table 4), despite there being a trend for higher R_Sym_ in the *E. pallida*-*S. minutum* holobiont. There was, however, a significant negative correlation between host- and symbiont specific CS activities in this particular symbiosis (Pearson's correlation test: *r* = −0.708, *t* = −4.139, df = 17, *p* = 0.0006). Median values for 1-ß for anemones hosting *Symbiodinium* A4 and *S. minutum* were similar at 0.08–0.09 (ranges: 0.02–0.16 and 0.04–0.14, respectively). However, the relationship between 1-ß and R_Sym_ differed between holobionts, with the slope close to unity for *E. pallida*-*S. minutum* symbioses, but significantly lower for the *E. pallida*-*Symbiodinium* A4 holobiont [Figure 4B; Linear regression: *F*_(3, 34)_ = 18.04, *p* < 0.001; *t*_(1−β × holobiont)_ = 3.404, *p* = 0.002]. These differences were mirrored in the R_Sym_/1-ß ratios of individual anemones, which were significantly higher in animals hosting *S. minutum* (Table 4, Figure 5F).

**Table 4:** **Comparison of respiratory and photosynthetic parameters in experimentally re-infected *Exaiptasia pallida* anemones**.

**Variable**	**Test statistic**	***p***
Symbiont density (cells mg^−1^)^‡^	*t* = 2.289	**0.028**
Photosynthetic output (μmol O_2_ h^−1^ cell^−1^)	*t* = −2.701	**0.010**
Holobiont respiration rate (μmol O_2_ h^−1^ mg^−1^)^‡^	*t* = 2.329	**0.026**
Host citrate synthase activity (U mg^−1^)	*t* = −1.995	0.054
Symbiont citrate synthase activity (U mg^−1^)^†^	*W* = 79	**0.002**
R_Sym_ (dimensionless)^†^	*W* = 128	0.130
R_Sym_/1-ß(dimensionless)	*t* = −2.615	**0.013**

*Bold values indicate statistical significance at p < 0.05*.

*R_Sym_ and 1-ß are as defined in the text*.

No symbol: t-test on raw data, df = 36;

† *Wilcoxon Rank Sum test*,

‡ *t-test on log_10_-transformed data, df = 36*.

## Discussion

It remains unclear how the metabolic activities of the animal host and its algal symbionts interact to determine the physiology of the symbiotic cnidarian “holobiont” (Yellowlees et al., 2008; Davy et al., 2012). Specifically, we have a limited understanding of the partitioning of respiration between host and symbiont *in hospite*. Yet, quantifying algal respiration within the animal is critical to the study of carbon/energy transfer between symbiotic partners (Muscatine et al., 1981, 1983; Steen and Muscatine, 1984; Davy et al., 1996, 2012). By measuring the activity of a key mitochondrial enzyme in the animal and its algal symbionts, this investigation represents a significant step forward in our understanding. In particular, to the best of our knowledge, this is the first time that the aerobic capacities of two algal symbiont species have been directly compared in the same host.

### mtDNA copy number as an indicator of mitochondrial density

For some organisms, the ratio between mitochondrial- and nuclear genomes serves as a proxy for mitochondrial density and, hence, aerobic capacity (e.g., Ballard and Melvin, 2007; Urschel and O'Brien, 2008). However, the mtDNA/nucDNA ratio was not a useful predictor of *E. pallida* respiration rate (as measured by O_2_ consumption) or aerobic capacity (as measured by citrate synthase activity). The high variability of mtDNA/nucDNA ratios was surprising. However, if mitochondrial copy number ratio is heritable (Ding et al., 2015), this variability may reflect a high genetic diversity within our *E. pallida* population. Preliminary analysis of microsatellite *loci* as described by Hoadley et al. (2015) suggested that at least 60% of our anemones were genetically distinct individuals (data not shown). Likewise, anemone size was also highly variable in our natural population (range 0.1–2.0 mg total soluble protein) and mtDNA/nucDNA can vary with age (Simonetti et al., 1992; Barazzoni et al., 2000). Further work is clearly needed before we can conclude whether mtDNA copy number is a useful marker of mitochondrial density in anthozoans. Declining mtDNA/nucDNA ratio can indicate oxidative stress-induced mtDNA damage (Liu et al., 2003; Ballard and Melvin, 2007; Hunter et al., 2010), and this might be a useful application of the technique in symbiotic cnidarians, where oxidative stress can be a significant component of physiological dysfunction (Weis, 2008).

### Partitioning of respiration between symbiotic partners

#### Variability in a naturally symbiotic sea anemone population

We noted two main trends in using CS activity as a marker of aerobic respiration in a natural population of *E. pallida*. Firstly, protein-normalized holobiont respiration rates and host CS specific activity decreased with increasing anemone biomass in a manner similar to that reported by Verde and McCloskey (1996) and Thuesen and Childress (1994) in benthic and planktonic scyphozoans. We also found positive associations between symbiont density and both holobiont respiration rate and host CS specific activity. A positive relationship between holobiont respiration rate and increasing algal symbiont density is common in symbiotic cnidarians (Hoegh-Guldberg and Hinde, 1986; Hoogenboom et al., 2010; Starzak et al., 2014), although not ubiquitous (see Hoegh-Guldberg and Smith, 1989). This has been interpreted as the increasing contribution of algal symbiont respiration, and has been used to estimate algal respiration rate *via* regression analysis (Hoogenboom et al., 2010 and references therein). However, this interpretation assumes a constant rate of animal host respiration across changing algal densities. The coupling of host CS activity with symbiont density suggests that such an assumption would be incorrect for the *E. pallida* used here. Agostini et al. (2013) reported a positive correlation between symbiont density and holobiont electron transport system activity and hypothesized that this reflected an increased availability of respiratory substrates. We might also expect host respiration to increase with symbiont density due to demands on host carbonic anhydrase to provide inorganic carbon to the algae (Weis, 1993; Hopkinson et al., 2015). Equally, higher host respiration rates could promote symbiont population growth through elevated tissue CO_2_ concentrations (Wooldridge, 2014). Regardless of the mechanistic link between holobiont respiration and algal symbiont density, our findings mean that correlations between these two variables should be interpreted with caution, since they likely reflect changes in the respiration of both symbiotic partners.

The results presented here have implications for the generality of one of the central assumptions for calculating CZAR—the contribution of *Symbiodinium* to animal respiration. Specifically, *in-vivo* respiration rates of animal and alga are assumed to be proportional to their respective biomasses (measured as protein content and designated ß and 1-ß, respectively) (Muscatine et al., 1981, 1983), and this proportionality is generally assumed to be 1:1 (e.g., Fitt and Pardy, 1981; Muller-Parker, 1984; Steen and Muscatine, 1984; Starzak et al., 2014). However, we noted a positive association between anemone specific CS activity and *Symbiodinium* cell density, and furthermore, a significant negative correlation between animal- and algal specific CS activities. Together, these findings suggest that as the proportion of symbiont biomass increases, the contribution of algal symbiont CS to holobiont CS (i.e., the “symbiont respiration fraction,” defined here as R_Sym_) might increase at a slower rate. Indeed, in our natural *E. pallida* population, R_Sym_ was 31% [±20% (95% CI)] lower than would have been expected from the assumption R_Sym_ = 1-ß. Such an assumption would in this instance lead to overestimation of algal symbiont respiration (and vice versa for the animal host), potentially confounding the analysis of organic carbon translocation from symbiont to host. In short, the finding that R_Sym_≠ 1-ß questions the validity of studies applying the growth rate method to determine carbon budgets in animal-algal symbioses (Muscatine et al., 1981, 1983; Falkowski et al., 1984; Verde and McCloskey, 1996; Levas et al., 2013; Starzak et al., 2014).

#### Effect of holobiont composition on the partitioning of respiratory activity between symbiotic partners

Respiration rates vary between different holobionts (Verde and McCloskey, 1996; Agostini et al., 2013; Starzak et al., 2014; Hoogenboom et al., 2015) and, in our re-infected *E. pallida*, were slightly lower (*ca*. 10%) in animals hosting *S. minutum* than in animals associating with *Symbiodinium* A4. To some degree this must reflect a lower *S. minutum* population density within the host, which itself likely reflects the heterologous nature of the *S. minutum* symbiont. Photosynthesis per alga, however, was higher in *S. minutum* than in *Symbiodinium* A4 and, ordinarily, this would be interpreted alongside holobiont respiration, algal biomass and growth rates, in order to calculate CZAR and estimate symbiont-host carbon flux. Yet, higher CS activity in *S. minutum* suggests that this symbiont species was both respiring and photosynthesizing to a greater degree than was *Symbiodinium* A4. Thus, one should be cautious in interpreting higher rates of photosynthetic O_2_ evolution in *S. minutum* as evidence for a greater pool of fixed carbon available for translocation to the host.

Returning to the assumption R_Sym_ = 1-ß (Muscatine et al., 1981), we noted that values for 1-ß were similar for anemones hosting *Symbiodinium* A4 and *S. minutum*. That this is the case despite the lower symbiont densities in the latter association might be due to slightly, but not significantly, higher protein content per cell in *S. minutum* [mean: 38 ± 4 (SE) pg cell^−1^] relative to *Symbiodinium* A4 [29 ± 4 (SE) pg cell^−1^]. The effect of the near-equivalency of 1-ß in the context of the symbionts' differing aerobic capacities (measured as CS activity) is that the relationship between R_Sym_ and 1-ß differs significantly between the two holobionts (Figures 4B, 5F). In short, for the same proportion of biomass, *S. minutum* accounts for more of the total aerobic capacity of the holobiont than does *Symbiodinium* A4.

### Limitations of a biochemical approach to quantifying respiration

We acknowledge the limitations in using metabolic enzymes as markers of respiration in an endosymbiosis. While we have demonstrated that the adequate separation of animal host and algal symbiont material is possible when working with *E. pallida*, applying these techniques to reef corals could be more challenging due to high mucus contents and the presence of an internal skeleton. A further limitation is the specificity of the selected enzyme(s), as many mitochondrial enzymes have secondary roles that are unrelated to respiration (e.g., fumarase is involved in cytosolic responses to DNA damage; Yogev et al., 2010). We cannot discount the influence of alternate metabolic pathways on the CS activities observed here, but the strong association between CS activity and O_2_ consumption suggests that this enzyme remains a useful marker for respiratory function. Reliable enzyme assays require that the enzyme-catalyzed reaction is carefully controlled and usually enzyme-limited. However, under natural conditions the metabolic enzymes of marine invertebrates are often present in excess so that the organisms can respond rapidly to changes in energy needs, trophic state, or the surrounding environment (Båmstedt, 1980; Martinez-Cruz et al., 2012). Thus, the same reaction *in-vivo* is more likely to be substrate-limited. Concretely, changes in CS activity as measured here reflect changes in the density of mitochondria or in the CS-contents of individual mitochondria (Spinazzi et al., 2012). The CS assay as applied here cannot detect short-term changes in respiration rate such as those resulting from the transfer of a photosynthetic organism from the dark to the light. Therefore, our data do not address the first assumption of the CZAR calculations—that animal host respiration rate in symbiotic cnidarians is unchanged upon illumination (Muscatine et al., 1981).

### Summary and conclusions

Here we describe a novel approach to addressing the question of how respiratory activity is partitioned between animal host and algal symbiont in the cnidarian-dinoflagellate association. Our quantification of mitochondrial genome copy number as a predictor of mitochondrial density was less informative than we expected, but such an approach is worthy of more attention in a clonal population or in individuals undergoing stress. By contrast, measurement of mitochondrial citrate synthase activity revealed interesting patterns in host- and symbiont aerobic capacity within and between different holobionts. Firstly, we can confirm a positive association between algal symbiont density and host aerobic capacity. Secondly, we noted that aerobic capacity of *Symbiodinium* cells *in hospite* was correlated negatively with that of their host and that different *Symbiodinium* species can have different aerobic capacities. To our knowledge, this is the first observation of differential *in-hospite* respiratory function between different *Symbiodinium* species in the same host animal. Perhaps most importantly, our data show that relying on symbiont biomass as the sole indicator of symbiont respiration can lead to inaccurate determination of respiratory partitioning within a single holobiont. Moreover, interspecific differences in algal respiration mean that this inaccuracy can vary between different holobionts. We therefore suggest that biochemical quantification of mitochondrial function should be considered when investigating respiratory function in different host-symbiont associations or under different environmental conditions.

## Author contributions

TH and MW conceived the experiments, hypotheses, and analytical methods. TH carried out the experimental work with assistance from JH and KH. TH, JH, and KH constructed the wet-lab facilities and maintained the anemone populations. KH constructed and programmed the custom-built LED-respirometry system. TH analyzed the data and wrote the manuscript. MW, JH, KH, and AM provided critical appraisal of the manuscript, statistical analyses and principal conclusions.

## Funding

This research was funded by the National Science Foundation (grant no. 1316055).

### Conflict of interest statement

The authors declare that the research was conducted in the absence of any commercial or financial relationships that could be construed as a potential conflict of interest.
