# Supplementary material for: Partitioning of Respiration in an Animal-Algal Symbiosis: Implications for Different Aerobic Capacity between Symbiodinium spp
Source: Front Physiol. 2016 Apr 18;7:128. doi: 10.3389/fphys.2016.00128 (PMC4834350; doi:10.3389/fphys.2016.00128)
Supplement: Supplementary file 1 [file DataSheet1.PDF]

ATP Synthase Subunit-6 (ATP-6) QPCR amplicon

```
1        10        20        30        40        50
|        |         |         |         |         |
CGTCTCGCCGCAAATTTATCAGCCGGCCTATTTATTATTTGCTATATTAG
CTGGGTTTGGCTTTAATATGTTAACCACAGCCAGGCGTCTTTAATATTTT
CCCTGTTTTGATTATGGTCTTTATAAGTCTACTAGAGGCCGCA
```

Cytochrome c Oxidase Subunit-1 (COI) QPCR amplicon

```
1        10        20        30        40        50
|        |         |         |         |         |
GTCTCCCAGCCGGAAATAAAAATTGGAATAAAGATGTTTACTACAACTAC
TCAGGTTTTAAGATCCTCAGAGACTGCATGCGAGAGATGCTGACAATGGT
```

Eukaryotic Translation Elongation Factor 1-alpha (EF-1-a) QPCR amplicon

```
1        10        20        30        40        50
|        |         |         |         |         |
GCACTGAGCCACCATACAGCGAGCCCCGATTCAACGAAATCAAGAAAGAA
GTATCAAATTTCTTGAAGAAGACCGGCTATAACCCAAA
```
