# Supplementary material for: Partitioning of Respiration in an Animal-Algal Symbiosis: Implications for Different Aerobic Capacity between Symbiodinium spp
Source: Front Physiol. 2016 Apr 18;7:128. doi: 10.3389/fphys.2016.00128 (PMC4834350; doi:10.3389/fphys.2016.00128)
Supplement: Supplementary file 2 [file DataSheet2.PDF]

ATP Sythase Subunit-6 (ATP-6) cloned standard

```
1         10        20        30        40        50
|         |         |         |         |         |
GGTTGTTACGTTAGGCTTGTCATTTTCAATTGTGGTAGGTGTAACTTTAG
GTGGACTTTGGAAATTTAAATGGAACTTTTTAAGTATTTTAATGCCAGCT
GGGGCCCCCTTAGCATTAGCTCCTCTTTTAGTATTAATTGAAACAGTAAG
TTATATATCAAGAGCTATCTCTTTAGGGGTCCGTCTCGCCGCAAATTTAT
CAGCCGGCCTATTTATTATTTGCTATATTAGCTGGGTTTGGCTTTAATAT
GTTAACCACAGCCAGGCGTCTTTAATATTTTCCCTGTTTTGATTATGGTC
TTTATAAGTCTACTAGAGGCCGCAGTGGCGGTTATTCAAGC
```

Cytochrome c Oxidase Subunit-1 (COI) cloned standard

```
1         10        20        30        40        50
|         |         |         |         |         |
AGCCGTCAGAGACAGTAATGTTTTTGTTTATTATCTCGCTATATGCTGGA
AAAACCCTCTTTAAAATAAGTGCTCATCTAAGGTTAAGATAGCAAAATCT
TATTTTACAAGGTCTCCCAGCCGGAAATAAAAATTGGAATAAAGATGTTT
ACTACAACTACTCAGGTTTTAAGATCCTCAGAGACTGCATGCGAGAGATG
CTGACAATGGTTAATTGGGTTAATTGAAGCTAAAAGTTGCTTAAATGTAG
CTCGAAAATCTTATAATATAGAAAGGCTCTCTCTTTCTATTTCTTGTTCC
CTTAGAAATATTCAGGTTCTCTACTACATAAAAGGTTTATTAGGGCTTGG
TCACG
```
