# Supplementary material for: Partitioning of Respiration in an Animal-Algal Symbiosis: Implications for Different Aerobic Capacity between Symbiodinium spp
Source: Front Physiol. 2016 Apr 18;7:128. doi: 10.3389/fphys.2016.00128 (PMC4834350; doi:10.3389/fphys.2016.00128)
Supplement: Supplementary file 3 [file DataSheet3.PDF]

Eukaryotic Translation Elongation Factor 1-alpha (EF-1-a) cloned plasmid

```
1         10        20        30        40        50
|         |         |         |         |         |
AATTGACAGCACTGAGCCACCATACAGCGAGCCCCGATTCAACGAAATCA
AGAAAGAAGTATCAAATTTCTTGAAGAAGACCGGCTATAACCCAAAAGCT
GTCATCTTTGTACCAATCTCAGGATGGCACGGTGATAACATGATTGAACC
ATCGACAAAGATAAGTTGTGAACAATAGACCACTTTCATAAGTACCTTGC
ATCGATTTTATATAAATGACAATGTGGAAACGTTTGACACCATTATATGG
AAGAACTCTCAGAATTATATATTTTATAAGTTTTAACACTGAAGTTATGG
AGGGATTCAAGATACAAGTATTTGTATGAAATTCTTTGCTAAGGCCTAGG
TAGACTTGTTTTAGTGTTTTTGTGCCTTTTCTAACAATTATTGATGCAAG
AGGATTCAATATTTTTTCTCTGTCTATCATCTGACTGTTTTTTTTTTGTC
ATTATAACATTATGAATTTATCGATTATATGCTAACTAGACGCTTGTAAA
GCATGATTCGGGCTAGCCTTTTTGGAAAGTTGAGAAAGACTGGATATTAC
ATGGGTGCGCTAATACACCAAGAGCAAAATATTTTCAATACAATGAGCTT
ACACAATCTCTGAGTTAAAAATCGATGATTTTTTTTTTGAACAGGATTTG
AACCCATGACCTTTGCGATATACTGCATATACTGGTTTTGAAATTGCAAT
CATTGTTACTTTGCAGATGCCTTGGTTCAAGGATGGACTGTTGAACAGG
TCTTAGAAAAAGGCAAGGAACCCARGTTTCAAAGGGCAACACCCTCCTAG
AAG
```
