# Supplementary figures and images for: Partitioning of Respiration in an Animal-Algal Symbiosis: Implications for Different Aerobic Capacity between Symbiodinium spp
Source: Front Physiol. 2016 Apr 18;7:128. doi: 10.3389/fphys.2016.00128 (PMC4834350; doi:10.3389/fphys.2016.00128)

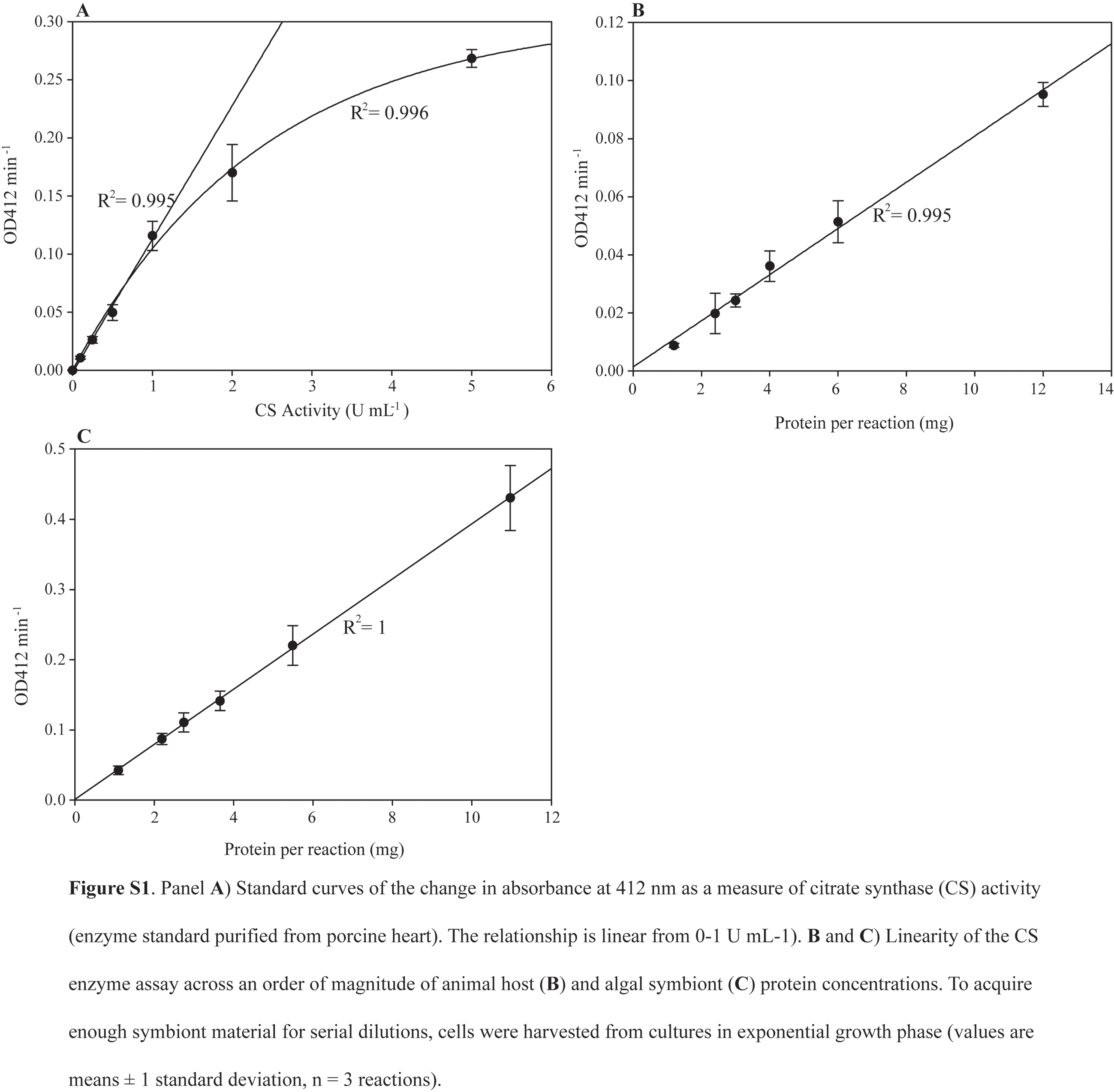

Supplement: Supplementary file 4 [file Image1.TIF]

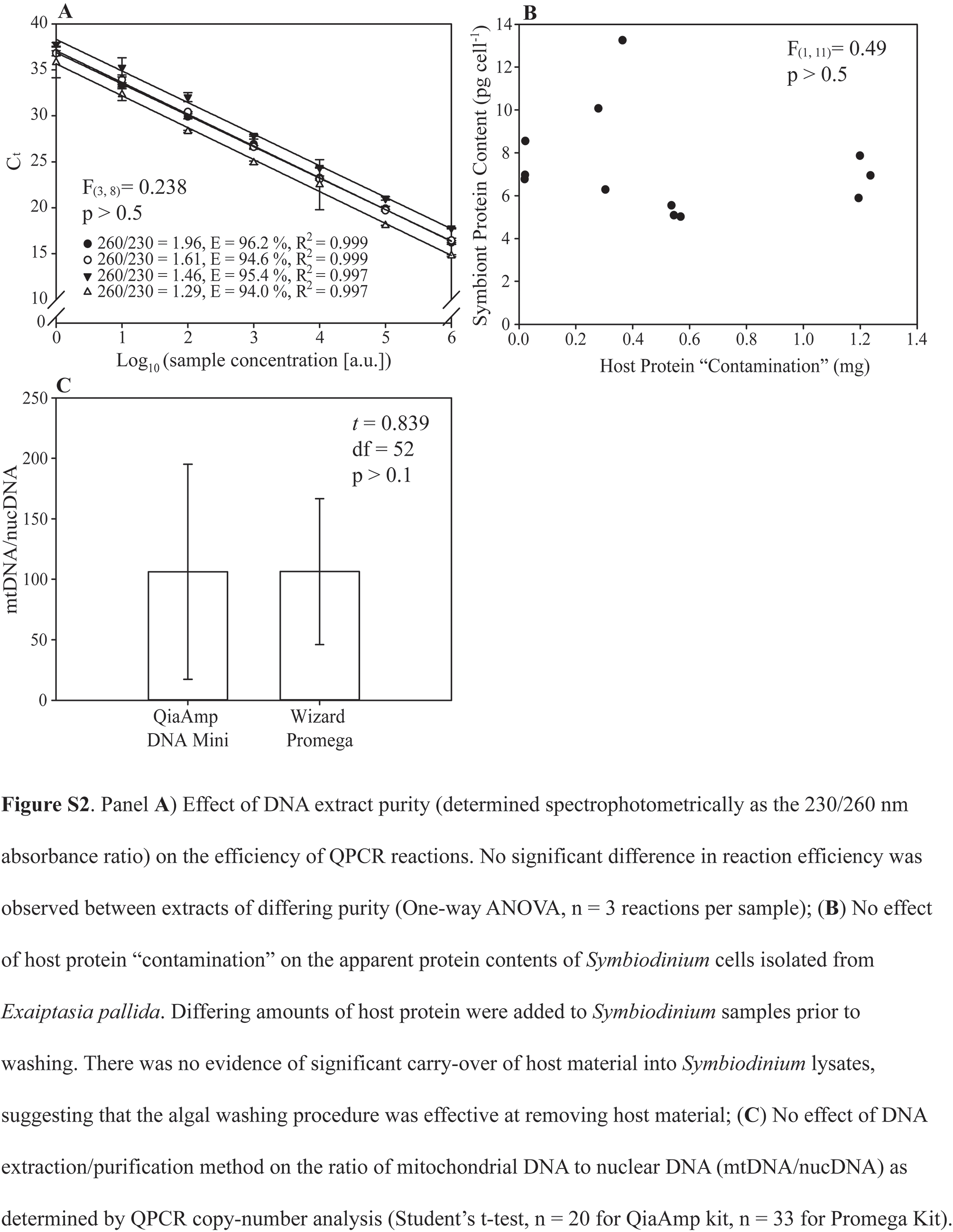

Supplement: Supplementary file 5 [file Image2.TIF]

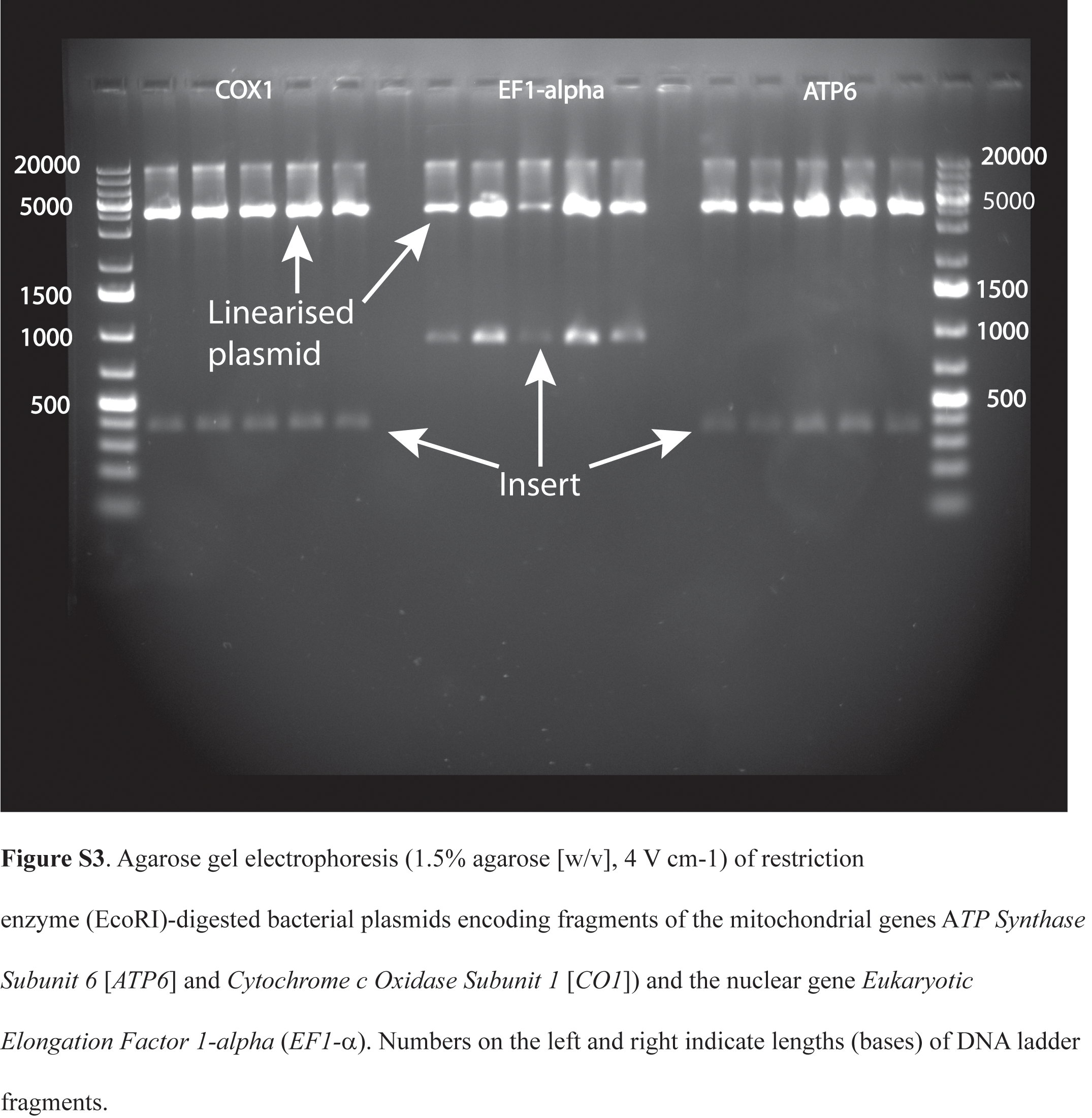

Supplement: Supplementary file 6 [file Image3.TIF]
